# Postoperative complications are main reason for noncompliance with enhanced recovery after surgery program in patients undergoing hepatectomy and pancreatectomy

**DOI:** 10.1002/jgh3.12250

**Published:** 2019-08-27

**Authors:** Justus Philip, Richard Fairtile, Andrei Cocieru

**Affiliations:** ^1^ Department of Surgery Summa Akron City Hospital Akron Ohio USA; ^2^ Department of Anesthesiology Summa Akron City Hospital Akron Ohio USA; ^3^ Northeastern Ohio Medical University Roostown Ohio USA

**Keywords:** compliance, enhanced recovery after surgery, hepatectomy, pancreatectomy

## Abstract

**Background and Aim:**

Enhanced recovery after surgery (ERAS) protocols are reported to improve postoperative outcomes in patients undergoing a routine protocol and postoperative outcomes in patients undergoing hepatic and pancreatic resections at our institution.

**Methods:**

A total of 99 consecutive patients at a single institution managed with a similar ERAS protocol were divided into the “early” (50 patients) and “late” (49 patients) cohorts. Both cohorts were statistically identical in demographics and range of surgical procedures performed. Postoperative complications, readmission, reoperation rates, and length of stay were analyzed. Categorical variables were statistically compared using Fisher's exact test and continuous variables using *t*‐test and Mann–Whitney U‐test when appropriate.

**Results:**

There were 32 hepatectomies/18 pancreatectomies in the “early” cohort and 22 hepatectomies/29 pancreatectomies in the “late” cohort. The overall complication rate was 38.8%, with a 30‐day readmission rate and reoperation rate of 16.1 and 5%, respectively. There was one mortality (1%). Group‐specific overall complication rate (40 *vs* 38.7%, *P* = 0.8), readmission rate (20 *vs* 12.2%, *P* = 0.4), reoperation rate (6 *vs* 4%, *P* = 1.0), and mortality (2 *vs* 0%, *P* = 1.0) were not statistically significant between both groups.

**Conclusions:**

Despite similar rates of adherence to the established ERAS 24 protocol, there was no improvement in median length of stay (7 days) between the “early” and “late” groups. The only reason for noncompliance with the ERAS protocol was development of surgery‐related complications.

## Graphical abstract

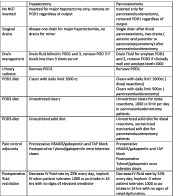

Enhanced recovery after surgery (ERAS) programs are used to improve postoperative outcomes such as lengths of stay and complications after hepatobiliopancreatic surgery. Logistic barriers in program implementation, as well as postoperative complications, are cited as the main reasons for noncompliance with ERAS. In our study, only postoperative complications were the main reason for noncompliance with the ERAS protocol.

## Introduction

Recently, multiple publications have demonstrated the feasibility and safety of enhanced recovery after surgery (ERAS) protocols in hepatobiliopancreatic (HPB) surgery.^1^, ^2^, ^3^, ^4^ Postulated benefits of ERAS included decrease in length of stay and hospital cost without a negative impact on perioperative mortality and morbidity or readmission rate.^1^, ^2^, ^3^, ^4^ There is significant variability in reported outcomes, and the validity of conclusions is usually based on low‐quality evidence.^5^ What is clear is that compliance with and audit of the established protocol appears to be an important element in the implementation of a successful ERAS program.^6^ We decided to study the relationship between compliance with established HPB ERAS protocol and perioperative outcomes at our institution.

## Methods

This study was performed as an analysis of a prospectively kept database of all patients undergoing routine liver and pancreatic resections carried out consecutively at our institution from August 2013 until December 2017 by a single surgeon (Andrei Cocieru). Inclusion criteria included all patients who were at least 18 years old and were deemed to be fit to undergo surgery during preoperative evaluation. The ERAS protocol was introduced in a stepwise fashion and included analysis of current literature, formulation of the ERAS protocol, discussion with nursing and resident staff, and monitoring of compliance by implementing key elements of the protocol (Table 1) using daily rounds and an electronic order system documenting completion of the physician order. All perioperative outcomes and implementation of ERAS protocol key elements were recorded in an electronic database. The study cohort was divided into an “early” group of 50 patients in which feasibility of ERAS program elements was tested and a “late” group of the subsequent 49 patients in which the same ERAS protocol was used without any modifications. Division was based on previously reported number of cases needed for resident and nursing staff to familiarize themselves with key elements of the program. Program key elements were based on current recommendations of ERAS society with some modifications.^7^, ^8^ Compliance was defined as implementation of those key elements and was monitored, along with reasons for noncompliance, which were recorded in the database. Compliance with ERAS protocol was considered incomplete if one or more elements of the protocol were not implemented for any reason. Major hepatectomy was defined as resection of more than three segments. Both groups were compared by age, gender, diagnosis, body mass index, American Society of Anesthesiologists (ASA) class, blood loss, and length of surgery as expressed in mean and median values where appropriate. Length of surgery was recorded from the time the patient arrived at the operating room to the time the patient left the operating room. Postoperative outcomes analyzed included length of stay, morbidity and mortality, and reoperation and readmission rates. Mortality, morbidity, and readmission rates were calculated for period of hospital stay or 30 days postoperatively according to the Clavien‐Dindo classification.^9^ HPB‐specific complications were defined by the International Study Group in Pancreatic and Liver Surgery.^10^, ^11^, ^12^, ^13^, ^14^, ^15^ Categorical variables were statistically compared using Fisher's exact test and continuous variables using *t*‐test and Mann–Whitney U‐test using GraphPad Quick Calcs software. Statistical significance was set at *P* < 0.05. Informed consent for perioperative data collection was obtained from all patients. Institutional review Board (IRB) approval was obtained to conduct the current study.

**Table 1:** Enhanced recovery after surgery protocol

	Hepatectomy	Pancreatectomy
No NGT inserted	Inserted for major hepatectomy only, remove on POD 1 regardless of output	Inserted only for pancreatoduodenectomy, removed on POD 1 regardless of output
Surgical drains	Always one drain for major hepatectomies, no drains for minor	Single drain after distal pancreatectomy, two drains (anterior and posterior to pancreatojejunostomy) after pancreatoduodenectomy
Drain management	Drain fluid bilirubin on PODs 1 and 3, remove on POD 3 if levels less than three times that of serum	Drain fluid for amylase on PODs 1 and 3, remove on POD 3 if clinically well and amylase level < 1000
Urinary catheter	Remove on POD 1	Remove on POD 1
POD 1 diet	Clears with daily limit 1000 cc	Clears with daily limit 1000 cc (distal resections) Clears with daily limit 500 cc (pancreatoduodenectomy)
POD 2 diet	Unrestricted clears	Unrestricted clears for distal resections, 1000 cc limit per day in pancreatoduodenectomy patients
POD 3 diet	Unrestricted solid diet	Unrestricted solid diet for distal resections, unrestricted mechanical soft diet for pancreatoduodenectomy patients
Pain control adjuvants	Preoperative NSAIDS/gabapentin and TAP block Postoperative Tylenol/gabapentin once tolerates clears	Preoperative NSAIDS/gabapentin and TAP block Postoperative Tylenol/gabapentin once tolerates clears
Postoperative fluid restriction	Decrease IV fluid rate by 25% every day, heplock IV when patient tolerates 1000 cc postoperative intake in 24 h with no signs of elevated creatinine	Decrease IV fluid rate by 25% every day, heplock IV when patient tolerates 1000 cc postoperative intake in 24 h with no signs of renal dysfunction.

NGT, nasogastric tube; NSAIDS, non‐steroidal anti‐inflammatory medications; POD, postoperative day; TAP, transverse abdominis plane.

## Protocol description

All patients scheduled for elective cases were seen in the surgical clinic and by anesthesiology where details of the ERAS protocol (Table 1) were discussed and agreed upon. Preoperatively, clear fluids were allowed for up to 2 h before surgery. Every patient received a preset combination of drugs, which included 1000 mg of Tylenol, 300 mg of gabapentin, 20 mg of Pepcid, and 400 mg of Celebrex. In patient with significant liver dysfunction (cirrhosis or jaundiced patients), Tylenol was omitted. Celebrex and gabapentin were not used in patients older than 70 years of age. Preoperatively, all patients received mechanical thromboprophylaxis, and patients with minor liver or left‐sided pancreatic resections received an additional subcutaneous heparin injection. Preoperative antiemetics were not used routinely. In the operating room (OR), all patients underwent an ultrasound‐guided transverse abdominal muscle block by trained anesthesiology staff. No central line was routinely placed, and patients were managed with two large bore peripheral lines and an arterial line for blood pressure monitoring. Patients undergoing major hepatectomy were fluid restricted, and all the others were given fluids to achieve minimal urinary output of 0.5 mL/kg/h. Induction was achieved using a combination of propofol and rocuronium and was maintained with isoflurane and opioids. Body core temperature was maintained above 36 degrees Celsius and was monitored via an esophageal probe. For open cases, all major right‐sided hepatectomies were performed using a reversed J‐type incision, while all left‐sided, minor liver resections and pancreatectomies were performed via a midline laparotomy. In cases of laparoscopic surgery (minor hepatectomy or distal pancreatectomy), placement of ports was based on a specific procedure. All major hepatectomies and distal pancreatectomies were drained using a single 19 Fr Bard drain, while all pancreatoduodenectomies were drained with two 19 Bard drains, one anterior and one posterior to hepaticojejunostomy and pancreatojejunostomy. A nasogastric tube was not used in distal pancreatectomy or hepatectomy but was always placed in patients undergoing pancreatoduodenectomy. They were removed on POD 1 regardless of output. Surgical drain fluid was tested for bilirubin and amylase on PODs 1 and 3 and removed on POD 3 if the bilirubin level was less than three times the serum level, and amylase was below 1000 IU/mL in clinically stable patients. All patients were started on limited clears on POD 1 and were advanced as tolerated to solids by postoperative day 3 regardless of bowel function. All patients were mobilized out of bed on postoperative day 1 by a dedicated physical therapy nurse. Every patient without significant liver dysfunction received 1 g of Tylenol postoperatively every 8 h and received gabapentin, 300 mg in the morning and 600 mg in the evening, to aid with pain control. If no flatus was recorded on POD 3, all patients would routinely receive 30 cc of milk of magnesia. Fluid restriction was applied from POD 1 in the form of a progressive decrease of IV fluid by 25% on each consecutive day. Patients were deemed ready to be discharged when they were able to tolerate a regular diet, had full return of bowel function, and when pain was controlled with oral agents.

## Results

Patient groups were comparable in terms of age, gender, length of surgery, median blood loss, and type of procedure (Table 2). The early group had a higher prevalence of hepatectomy cases due to a greater number of minor hepatectomy cases, while the late group had a higher rate of pancreatectomies, but it did not reach statistical significance. Overall, there were 10 major hepatectomies (7 right, 2 right extended, and 1 left), 22 minor hepatectomies, and 18 pancreatectomies (9 pancreatoduodenectomies, 7 distal and subtotal pancreatectomies, 1 total pancreatectomy, and 1 enucleation) in the early group. The late group consisted of 22 hepatectomies with 10 major (8 right and 2 right extended) and 12 minor hepatectomies and 27 pancreatectomies (19 pancreatoduodenectomies and 8 distal and subtotal pancreatectomies). There was one postoperative mortality in the early group—a patient sustained massive myocardial infarction (MI) after distal pancreatectomy, with hemopericardium and cardiogenic shock, on postoperative day 14. Three patients in the early group underwent reoperation—one for small bowel obstruction, one for bile leak after pancreatoduodenectomy, and one required portal thrombectomy for portal vein thrombosis after right hepatectomy—resulting in a reoperation rate of 6%. No mortality was observed in the late group, but two patients required reoperation—one for debridement of deep wound infection and one for abdominal dehiscence after pancreatoduodenectomy (4% reoperation rate). No significant differences were noted in the number or class of postoperative complications or type of complications (medical *vs* surgical) in the both groups (Table 3, 4). Compliance with established protocol (all elements of ERAS protocol followed and implemented) was equally high in both groups—70 *versus* 74% (*P* = 1.0)—and this did not appear to affect outcomes. The only reason for a lack of compliance in our series was the development of postoperative surgical complications requiring alteration in management (such as oral intake restrictions in ileus, placement of NGT, starting total parenteral nutrition (TPN) or interventional radiology (IR) drain placement etc). Despite continuous adherence to ERAS protocol, no improvement in median length of stay between the early and late groups was noted (Tables 3, 4). There were no instances when the ERAS protocol element was not implemented because of a logistic or communication issue.

**Table 2:** “Early” *versus* “late” group comparison

	Early group, *n* = 50	Late group, *n* = 49	*P*‐value
Age, years (mean)/range, years	58.2/20–83	65/22–85	0.1
Gender, male *versus* female	24/26	19/30	0.4
ASA class (mean)	2.7	3	0.057
Length of surgery, min (median)/range, min	335/29–712	370/46–584	0.52
EBL, mL (median)/range, mL	500/20–2000	400/20–2600	0.91
Hepatectomy	32	22	0.07
Major	10	10	0.39
Minor	22	12	0.39
Combined*	6	3	1.0
Pancreatectomy	18	27	0.07
Benign *versus* malignant diagnosis	12/38	12/37	1.0
Open *versus* lap surgery	45/5	44/5	1.0

* Combined surgery indicates multiorgan resection.

ASA, American Society of Anesthesiologists; EBL, estimates blood loss.

**Table 3:** Main postoperative outcomes

	Overall	Early group, *n* = 50	Late group, *n* = 49	*P*‐value
Length of stay, days (median)/range, days	ND	7/2–22	7/1–20	0.3
Complication rate (%)	38.8	40	38.7	0.8
Medical *versus* surgical		14 *versus* 26	17.2 *versus* 21.5	1.0
Readmission rate (%)	16.1	20	12.2	0.4
Reoperation rate (%)	5	6	4	1.0
Mortality (%)	1	2 (1)	0	1.0
Adherence to ERAS protocol (%)		70 (35 out of 50 patients)	74 (36 out of 49 patients)	0.28

ERAS, enhanced recovery after surgery.

**Table 4:** Perioperative complications between the groups

Clavien class	Early group, number of complications	Later group, number of complications	*P*‐value
Class I	*n* = 8	*n* = 12	0.32
	Clostridium infection‐1 Urinary retention‐2 Diabetes insipidus‐1 Type A bile leak after hepatectomy‐2 Pleural effusion‐1 Atrial fibrillation‐1	Wound infection‐1 Biochemical pancreatic leak‐3 Type A bile leak after hepatectomy‐3 Pleural effusion‐2 Pneumonia‐1 Atrial fibrillation‐2	
Class 2	*n* = 4	*n* = 4	1.00
	Ileus requiring TPN/o NGT‐2	Ileus requiring TPN/NGT‐2	
	Pancreatic leak/DGE requiring TPN‐2	Pancreatic leak/DGE requiring TPN‐2	
Class 3a	*n* = 5	*n* = 1	0.20
	IR drainage of intra‐abdominal collection/leak‐4 IR drainage of pleural effusion‐1	IR drainage of intra‐abdominal collection/leak‐1	
Class 3b	*n* = 3	*n* = 2	1.00
	Reoperation for SBO‐1 Reoperation for bile leak‐1	Reoperation for deep wound infection‐1	
	Reoperation for portal vein thrombosis‐1	Reoperation for abdominal dehiscence‐1	

DGE, delayed gastric emptying; IR, interventional radiology; NGT, nasogastric tube; SBO, small bowel obstruction; TPN, total parenteral nutrition.

## Discussion

Current evidence and multiple publications support the role of ERAS protocols in decreasing the length of stay in both routine and complex surgeries, including HPB surgery.^6^ It is concluded that perioperative complications rates are not affected, some medical complications can be decreased, and length of stay is usually reduced without a negative impact on readmission and reoperation rates. There is no HPB‐specific research addressing questions of compliance and its impact on the success of ERAS protocols. A majority of papers published are on the topics of upper gastrointestinal (GI) and colorectal surgeries. In those studies, to improve outcomes, compliance with the protocol appears to be crucial. Better outcomes, including a decrease in complications, were noted when compliance continued to increase.^16^, ^17^ The available literature suggests that overall compliance rates range from around 60% to over 90%.^18^, ^19^, ^20^ The main reasons for noncompliance cited in the literature were development of postoperative complications or logistic and communication issues with protocol implementation.^21^, ^22^However, once stable compliance is achieved, it is unclear if an additional decrease in postoperative complications and length of stay is achievable. Length of stay is a complex result of not only patient readiness to be discharged but also hospital resources involved in discharge planning and the discharge process itself. In our study, we attempted to study the effect of ongoing adherence to the ERAS protocol in a group of patients undergoing routine pancreatic and hepatic resections at the same hospital. Division between each group was based on a previously reported number of patients necessary to achieve satisfactory adherence to the ERAS program at the institutional level.^23^ The main findings were that, despite the similar or improving compliance levels, ongoing use of the ERAS protocol did not result in the improvement of recorded perioperative outcomes and length of stay. Reasons for noncompliance were due to postoperative surgical complications precluding full implementation of all ERAS elements and not due to logistics or communication issues. Improving compliance therefore does not seem to be possible without decreasing postoperative surgical complication rates at our institution. Prevention of surgical complications appears to be the main target to be addressed in order to further improve postoperative outcomes.
